# Nutritional knowledge, food habits and health attitude of Chinese university students –a cross sectional study–

**DOI:** 10.1186/1475-2891-4-4

**Published:** 2005-02-09

**Authors:** Ruka Sakamaki, Kenji Toyama, Rie Amamoto, Chuan-Jun Liu, Naotaka Shinfuku

**Affiliations:** 1 International Center for Medical Research. Kobe University Graduate School of Medicine, Kobe, 650-0017, Japan; 2 Seinan Jo Gakuin University Faculty of Health and Welfare, Department of Nutritional Sciences, Kitakyusyu, 803-0835, Japan; 3 Department of Plastic Surgery, Kobe University Graduate School of Medicine, Kobe, 650-0017, Japan

## Abstract

**Background:**

We have previously shown that irregular lifestyle of young Japanese female students are significantly related to their desire to be thinner. In the present study, we examined the nutritional knowledge and food habits of Chinese university students and compared them with those of other Asian populations.

**Methods:**

A self-reported questionnaire was administered to 540 students, ranging in age from 19-24 years. Medical students from Beijing University (135 men and 150 women) in Northern China and Kunming Medical College in southern China (95 men and 160 women) participated in this study. The parametric variables were analyzed using the Student's *t*-test. Chi-square analyses were conducted for non-parametric variables

**Results:**

Our results showed that 80.5% of students had a normal BMI and 16.6 % of students were underweight with the prevalence of BMI>30 obesity being very low in this study sample. Young Chinese female students had a greater desire to be thinner (62.0%) than males (47.4%). Habits involving regular eating patterns and vegetable intake were reported and represent practices that ought to be encouraged.

**Conclusions:**

The university and college arenas represent the final opportunity for the health and nutritional education of a large number of students from the educator's perspective. Our findings suggest the need for strategies designed to improve competence in the area of nutrition.

## Background

The increasing problem of obesity has been observed in many lower-income countries during the last decades. China has adopted an open-market policy and experienced explosive economic growth, which has led to less food scarcity at the national level and to a remarkable transition in the structure of the diet of Chinese [1]. The composition of the Chinese diet has been shifting towards a diet higher in fat and meat, and lower in carbohydrates and fiber [2]. Additionally, decreased levels of physical activity and leisure are linked to increases in the prevalence of an overweight condition, obesity and diet-related non-communicable diseases [3].

In previous reports, we examined eating habits and dietary knowledge of female students in Japan. Our results showed that irregular lifestyle was significantly related to indefinite complaint, with the majority of students having a desire to be thinner although the prevalence of students who were overweight was very low in this study sample [4]. Universities and colleges are potentially important targets for the promotion of healthy lifestyles of the adult population. However, little is known concerning the body mass index (BMI) distribution and nutritional and health-related behavior of Chinese university students. The purpose of this study was to obtain a preliminary understanding of the relative level of BMI distribution of Chinese university students and to determine the nutritional knowledge and body-shape perceptions.

## Material and Methods

This study was carried out between February 2001 and April 2002. Medical students from Beijing University (135 men and 150 women) in Northern China and Kunming Medical College in southern China (95 men and 160 women) participated in this study. A sample of 540 students aged 19 – 24 years were administered a self-reported questionnaire. The questionnaire consisted of 21 questions regarding eating, drinking and smoking habits (19 questions), with 2 questions related to dieting (trying to lose weight). Self-reported height and weight were used to calculate BMI (kg/m^2^). The questionnaire was designed by the authors and based on a national dietary survey held by the Health and Labor Ministry of Japan. Some of the authors also traveled to China to investigate the dietary life of Chinese to facilitate questionnaire design. The questionnaire was first written in Japanese and then translated to Chinese utilizing fluent bilingual linguistic services. The translated Chinese version was back-translated to insure the original meaning was not lost. Informed consent was obtained from all participants of this study according to the Declaration of Helsinki. The statistical software package SPSS 10.0 was used for the analysis of data [5]. In this study, parametric variables were analyzed using the Student's *t*-test. Chi-square analyses were conducted for non-parametric variables. All analyses were two-tailed, and a 'p' value less than 0.05 was considered statistically significant.

## Results

### Characteristics of the sample and BMI categories

**Figure 1:**
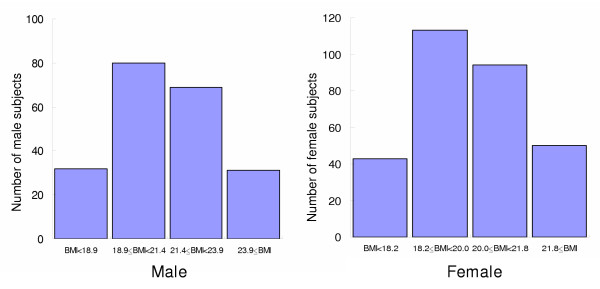
**BMI distribution of Chinese university students**. The BMI of male and female students was categorized into 4 groups according to mean BMI ± 1 standard deviation (SD).

The response rate was 96% (512 / 540). The characteristics of the subjects are shown in Table 1. A total of 212 men and 300 women, with a mean age of 20 ± 1.9 years, participated in this study. The average height was 165.8 ± 7.8 cm, while the average weight was 56.9 ± 9.2 kg. Mean BMI was 20.6 ± 2.2. To analyze the distribution of BMI and health-related behavior, BMI was categorized into 4 groups according to mean BMI of ± 1 standard deviation (SD) (Figure 1). The average BMI for male students was 21.4 ± 2.5 and was highest in the categories 18.9≤BMI<21.4 (37.7%) and 21.4≤BMI<23.9 (32.5%). The average BMI for female students was 20.0 ± 1.8, with the categories 18.2≤BMI<20.0 (37.5%) and 20.0≤BMI<21.8 (31.4%) displaying high values. According to WHO BMI classifications [6], 97.1% of students were classified into the underweight or normal weight categories. 2.5% (13/512) students were overweight (BMI>25) and 0.4% (2/512) of students were obese (BMI>30). BMI values of deviations from the average sample show the presence of few extreme values.

### Eating habit

The life style practices were compared by gender (see additional file 1). The majority of students (83.6 %) reported taking meals regularly, with 79.0% eating meals 3 times per day; there were no gender differences. However, a significant gender difference was found in the response relating to breakfast intake, with 66.8% of males and 82.3% of females reporting eating breakfast regularly (p < 0.0006). The frequency of snacking rate was significantly higher in females (31.1%) than in males (11.5%; p < 0.0001). The present sample demonstrated high consumption of vegetable and fruits. A total of 47.9% of students reported the consumption of colored vegetables such as spinach and carrots, and 32.5% of subjects reported eating fruit daily. Female students tend to eat more fruit than males (p < 0.0001). In addition, female students tend to eat with friends and family more frequently than males (p < 0.01). Few subjects smoke or drink alcohol. When the students eat out, female students are more likely to consider the calorie content of the menu than males (data not shown). Although 85.6% of students are aware of the concept of nutritionally balanced food, only a small number of students (7%) apply this concept when selecting food from a menu. Moreover, only 51% of students showed a desire to learn about healthy diets.

### Body image and health consciousness

When subjects were asked about their history of dieting, 22.7% of respondents reported that they had dieted (see additional file 2). The proportion of female students having a dieting experience (29.8%) was more than twice as great as that of male subjects (12.7%; p < 0.0006). In total, 56% of the students selected 'thin or slim is beautiful'. The percentage by gender was, 47.4% for male and 62.0% for female students. Female students have a significantly greater desire to be thinner than males (p < 0.001). More than half of the respondents reported a desire to adopt healthier dietary habits. Moreover, a question regarding the degree of consciousness pertaining to health and diet was asked; 45.2% of male students and 48.3% of female students wish to learn about health and diet. Among female subjects, BMI<18.2 strongly showed their consciousness of health and diet (p < 0.03).

## Discussion

This study aimed to determine the health, nutritional knowledge and dietary behavior of university students in China. As a result, we recorded the distribution of BMI among Chinese students and found a low prevalence of obesity, a finding that is consistent with a study of Japanese female students (BMI≥25 overweight was 5.8%, BMI>30 of obesity was 0%) [4]. In the United States, 35% of the college students are reported to be overweight or obese (BMI≥25) [7]. According to the WHO definition of obesity, BMI>30 is the cut-off point [6]. The definition is based on research of Caucasian populations. Asian populations are reported to have a higher body fat (%) at a lower BMI compared to Caucasians [8]. The WHO expert consultation reported that BMI in Asian populations is related to disease at a lower level [9]. In order to compare obesity prevalence between ethnic groups, BMI cut-off points for Asians need to be considered by well constructed and standardized body composition studies. It is notable that in China, the prevalence of overweight individuals increased from 1991 to 1997, with the increasing rate changing from 6.4 to 7.7 [10]. The proportion of energy derived from the fat of both vegetable and animal sources increased each year. A recent study revealed that energy derived from dietary fat accounted for more than 30% of the total energy [11]. Changes in dietary composition, which correspond to socioeconomic growth, may accelerate the prevalence of obesity in China.

The results of our study show that the majority of students regularly eat three times per day, and almost 80% of students eat vegetables and fruit twice per day. These eating habits ought to be encouraged. The traditional Chinese diet contains plenty of vegetables and is rice-based. The present study reported a high proportion of Chinese students eat breakfast daily. In contrast, a dietary survey of young Japanese subjects revealed a low rate of individuals engaged in regular eating patterns [12]. The skipping of breakfast has been associated with lower nutritional status and the risk of cardiovascular diseases [13]. It has also been reported that less adequate breakfast habits may contribute to the appearance and further development of obesity [14]. Therefore the importance of regular eating patterns cannot be overemphasized in nutritional education.

Our results showed that body figure perception was significantly different between female and male students. A number of researchers have investigated the relationship of body image and gender role. Women tend to desire a thinner figure, express more anxiety about becoming fat, and are more likely to diet than men [15,16]. In contrast, men have reported a desire for a heavier physique and muscularity [17]. In recent years, eating disorders have been increasing dramatically among young women. The results of our study did not confirm this suggestion to the level of statistical significance; however, it is worth pointing out that 65.0% of female students with BMI<20, which is under to normal weight range, indicated a desire to be thin. Dissatisfaction with body figure and eating disorders are closely related [18-20]. Being young, female, and dieting are identified risk factors that have been reliably linked to the development of eating disorders [21]. It was speculated that some of the students who were preoccupied with a thin body may develop eating disturbances. Thus, the promotion of healthy weight management practices should be considered when developing health education programs.

## Conclusions

In conclusion, our findings reveal that the majority of students were classified into the normal BMI group, with the prevalence of BMI >30 obesity being very low in this study sample. Young female students had a greater desire to be thinner than male students. Habits involving regular eating patterns and vegetable intake were found and represent practices that ought to be encouraged. The meal and snack patterns in Chinese students were very similar to the traditional eating pattern model, although diets are changing rapidly in China and other low-income countries. The university and college arenas represent the final opportunity for nutritional education of a large number of students from the educator's perspective. Our findings suggest the need for strategies designed to improve competence in the area of nutrition, especially with respect to information relating to sources of nutrition and healthy weight management. Furthermore, public demand for health and nutritional information should be taken into consideration when implementing strategies aimed at improving the nutritional well-being of individuals.

## Authors' contributions

R.S carried out questionnaire design, manuscript drafting and total coordination of the study. K.T has been involved in drafting and revision of the article. R.A contributed to the data entry and its analysis. L.CJ contributed to the questionnaire design, data collection and language translations. N.S contributed to final approval of the manuscript.

## Supplementary Material

Additional File 1 Table 2 containing the results of questions related to lifestyle practices with special reference to food habit. The meal patterns, consumption of fruits and vegetables, consumption of fried foods, consumption of alcohol were assessed in male and female students. The Chi-square analyses were employed to compare the behavioral differences by gender. The evaluations of statistical significance were made at the p < 0.05. Click here for file

Additional File 2 Table 3 contains the results of body shape perception and health consciousness of male and female students. Male and female respondents were categorized in to 4 groups respectively, according to mean BMI of ± 1 standard deviation (SD). Analyses were made between BMI groups using Chi-square analysis. The evaluations of statistical significance were made at the p < 0.05. Click here for file

## Figures and Tables

**Table 1:** Characteristics of Participants

	**Total**	**Male**	**Female**
**Variable**	n = 512	n = 212	n = 300
**Age (y)**	20.4 ± 1.9	20.3 ± 1.7	20.4 ± 2.0
**weight (kg)**	56.9 ± 9.2	63.7 ± 8.8	52.1 ± 5.9
**height (cm)**	165.8 ± 7.8	172.3 ± 5.5	161.2 ± 5.6
**BMI (kg/m^2^)**	20.6 ± 2.2	21.4 ± 2.5	20.0 ± 1.8

Gender distribution in the sampled population. BMI is based on self-reported height and weight. BMI = weight [kg] / height [m]^2^
