# Supplementary material for: Nutritional knowledge, food habits and health attitude of Chinese university students –a cross sectional study–
Source: Nutr J. 2005 Feb 9;4:4. doi: 10.1186/1475-2891-4-4 (PMC553986; doi:10.1186/1475-2891-4-4)
Supplement: Additional File 1 — Table 2 containing the results of questions related to lifestyle practices with special reference to food habit. The meal patterns, consumption of fruits and vegetables, consumption of fried foods, consumption of alcohol were assessed in male and female students. The Chi-square analyses were employed to compare the behavioral differences by gender. The evaluations of statistical significance were made at the p < 0.05. [file 1475-2891-4-4-S1.doc]

Table 2- Lifestyle practices by gender

| **Questions** | **Levels** | **Total (%)** | | **Male (%)** | | **Female(%)** | | **p values** |
| --- | --- | --- | --- | --- | --- | --- | --- | --- |
| Do you take your meals regularly | always regular | 428 | (83.6) | 173 | (81.6) | 255 | (85.0) | n.s |
|  | irregular | 84 | (16.4) | 39 | (18.4) | 45 | (15.0) |  |
| Do you always take breakfast | daily | 387 | (75.9) | 141 | (66.8) | 246 | (82.3) |  |
|  | three or four times per week | 56 | (11) | 29 | (13.7) | 27 | (9) | p<0.0006 |
|  | once or twice per week | 29 | (5.7) | 18 | (8.5) | 11 | (3.7) |  |
|  | rarely | 38 | (7.4) | 23 | (10.9) | 15 | (5) |  |
| How many times do you eat meals except snacks | one time | 4 | (0.8) | 1 | (0.5) | 3 | (1.0) | n.s |
|  | two times | 80 | (16) | 38 | (18.8) | 42 | (14) |  |
|  | three times | 395 | (79.0) | 150 | (74.3) | 245 | (82.3) |  |
|  | four times | 21 | (4.2) | 13 | (6.4) | 8 | (2.7) |  |
| How often do you take snacks apart from regular meals | daily | 117 | (23.1) | 24 | (11.5) | 93 | (31.3) | p<0.0001 |
|  | three or four times per week | 80 | (15.8) | 19 | (9.1) | 61 | (20.5) |  |
|  | once or twice per week | 148 | (29.2) | 58 | (27.8) | 90 | (30.3) |  |
|  | rarely | 161 | (31.8) | 108 | (51.7) | 53 | (17.8) |  |
| How often do you eat green, red or yellow colored vegetables | daily | 244 | (47.9) | 93 | (43.9) | 151 | (50.8) | n.s |
|  | three or four times per week | 190 | (37.2) | 84 | (39.6) | 106 | (35.5) |  |
|  | once or twice per week | 64 | (12.7) | 29 | (13.7) | 35 | (12.0) |  |
|  | rarely | 11 | (2.2) | 6 | (2.8) | 5 | (1.7) |  |
| How often do you eat fruits | daily | 166 | (32.5) | 56 | (26.4) | 110 | (36.8) | p<0.0001 |
|  | three or four times per week | 86 | (16.8) | 15 | (7.1) | 71 | (23.7) |  |
|  | once or twice per week | 138 | (27.0) | 54 | (25.5) | 84 | (28.1) |  |
|  | rarely | 120 | (23.5) | 87 | (41.0) | 33 | (11.1) |  |
| How often do you eat fried food | daily | 160 | (31.6) | 69 | (32.5) | 91 | (30.9) | n.s |
|  | three or four times per week | 55 | (10.8) | 25 | (11.8) | 30 | (10.1) |  |
|  | once or twice per week | 131 | (25.7) | 46 | (21.7) | 85 | (28.5) |  |
|  | rarely | 163 | (32.0) | 72 | (34.0) | 91 | (30.5) |  |
| How often do you take alcohol | daily | 4 | (0.8) | 2 | (1.0) | 2 | (0.7) | p<0.006 |
|  | two or three times per week | 23 | (4.6) | 17 | (8.1) | 6 | (2.1) |  |
|  | rarely | 473 | (94.6) | 191 | (91.0) | 282 | (97.3) |  |
| How often do you eat with friends and family | daily | 86 | (17) | 25 | (12) | 61 | (20.6) | p<0.01 |
|  | three or four times per week | 92 | (18.2) | 41 | (19.6) | 51 | (17.2) |  |
|  | once or twice per week | 165 | (32.7) | 63 | (30.1) | 102 | (34.5) |  |
|  | always alone | 161 | (31.9) | 80 | (38.3) | 81 | (27.7) |  |
| Please state your smoking history | current smoker | 36 | (7.0) | 29 | (10.3) | 7 | (0.7) | p<0.0001 |
|  | ex-smoker | 16 | (3.1) | 14 | (4.5) | 2 | (2.3) |  |
|  | never smoke | 459 | (89.8) | 169 | (85.2) | 290 | (97.0) |  |
| What type of food do you think you should eat to have a balanced nutrition | mainly meat | 14 | (2.8) | 10 | (4.8) | 4 | (1.4) | n.s |
|  | mainly vegetable | 40 | (7.9) | 13 | (6.3) | 27 | (9.1) |  |
|  | meat, vegetable and other variety of food | 438 | (86.9) | 178 | (85.6) | 260 | (87.8) |  |
|  | others | 12 | (2.4) | 7 | (3.4) | 5 | (1.7) |  |

The life style practices were compared by gender. Significant differences between sexes were determined by Chi-square analyses (p<0.05).
