# Supplementary material for: Nutritional knowledge, food habits and health attitude of Chinese university students –a cross sectional study–
Source: Nutr J. 2005 Feb 9;4:4. doi: 10.1186/1475-2891-4-4 (PMC553986; doi:10.1186/1475-2891-4-4)
Supplement: Additional File 2 — Table 3 contains the results of body shape perception and health consciousness of male and female students. Male and female respondents were categorized in to 4 groups respectively, according to mean BMI of ± 1 standard deviation (SD). Analyses were made between BMI groups using Chi-square analysis. The evaluations of statistical significance were made at the p < 0.05. [file 1475-2891-4-4-S2.doc]

Table 3 - Body shape perception and health consciousness of Chinese students

|  | **Male** | |  | |  | | | |  | | | |  | | | |  | | | | BMI categories | | | | | |  | | | |  | | | |  | | | |  | | | | |  | | | |  | | | |  |
| --- | --- | --- | --- | --- | --- | --- | --- | --- | --- | --- | --- | --- | --- | --- | --- | --- | --- | --- | --- | --- | --- | --- | --- | --- | --- | --- | --- | --- | --- | --- | --- | --- | --- | --- | --- | --- | --- | --- | --- | --- | --- | --- | --- | --- | --- | --- | --- | --- | --- | --- | --- | --- |
|  | **Questions** | | **Levels** | | **Total (%)** | | | | | | | | **< 18.9(%)** | | | | | | | | **18.9-21.4(%)** | | | | | | | | | | **21.4-23.9(%)** | | | | | | | | **≥23.9(%)** | | | | | | | | | **p values** | | | |  |
|  | Have you ever tried to be on a diet | | Yes | | | | 27 | | | | (12.7) | | | | 1 | | | | (3.1) | | | | 3 | | | (3.8) | | | | | | 8 | | | | (11.6) | | | | | 15 | | | | | (48.4) | | | p<0.0001 | | | |
|  |  |  | No | | | | 185 | | | | (87.3) | | | | 31 | | | | (96.9) | | | | 77 | | | (96.3) | | | | | | 61 | | | | (88.4) | | | | | 16 | | | | | (51.6) | | |  |  |  |  |
|  | Do you want to be slim to be beautiful | | Yes | | | | 100 | | | | (47.4) | | | | 15 | | | | (46.9) | | | | 41 | | | (51.9) | | | | | | 33 | | | | (47.8) | | | | | 11 | | | | | (35.5) | | | n.s | | | |
|  |  |  | No | | | | 111 | | | | (52.6) | | | | 17 | | | | (53.1) | | | | 38 | | | (48.1) | | | | | | 36 | | | | (52.2) | | | | | 20 | | | | | (64.5) | | |  |  |  |  |
|  | How often do you take snacks | | daily | | | | 24 | | | | (11.5) | | | | 4 | | | | (12.9) | | | | 12 | | | (15.2) | | | | | | 7 | | | | (10.3) | | | | | 1 | | | | | (3.2) | | | n.s | | | |
|  |  |  | three or four times per week | | | | 19 | | | | (9.1) | | | | 3 | | | | (9.7) | | | | 9 | | | (11.4) | | | | | | 5 | | | | (7.4) | | | | | 2 | | | | | (6.5) | | |  |  |  |  |
|  |  |  | once or twice per week | | | | 58 | | | | (27.8) | | | | 10 | | | | (32.3) | | | | 21 | | | (26.6) | | | | | | 19 | | | | (27.9) | | | | | 8 | | | | | (25.8) | | |  |  |  |  |
|  |  |  | rarely | | | | 108 | | | | (51.7) | | | | 14 | | | | (45.2) | | | | 37 | | | (46.8) | | | | | | 37 | | | | (54.4) | | | | | 20 | | | | | (64.5) | | |  |  |  |  |
|  | In general, how conscious you are of the | | I want to learn more | | | | 95 | | | | (45.2) | | | | 14 | | | | (43.8) | | | | 35 | | | (43.8) | | | | | | 31 | | | | (45.6) | | | | | 15 | | | | | (50.0) | | | n.s | | | |
|  |  |  | I want to learn in the future | | | | 85 | | | | (40.5) | | | | 14 | | | | (43.8) | | | | 33 | | | (41.3) | | | | | | 24 | | | | (35.3) | | | | | 14 | | | | | (46.7) | | |  |  |  |  |
|  |  |  | I am not interested in but better to lern | | | | 21 | | | | (10.0) | | | | 2 | | | | (6.3) | | | | 9 | | | (11.3) | | | | | | 9 | | | | (13.2) | | | | | 1 | | | | | (3.3) | | |  |  |  |  |
|  |  |  | I am not interested in this | | | | 9 | | | | (4.3) | | | | 2 | | | | (6.3) | | | | 3 | | | (3.8) | | | | | | 4 | | | | (5.9) | | | | | 0 | | | | | (0.0) | | |  |  |  |  |
|  | Do you consider you should change your | | I want to improve my dietary habits | | | | 111 | | | | (52.4) | | | | 14 | | | | (43.6) | | | | 40 | | | (50.0) | | | | | | 34 | | | | (49.3) | | | | | 23 | | | | | (74.2) | | | n.s | | | |
|  |  |  | I do not want to change | | | | 69 | | | | (32.5) | | | | 11 | | | | (34.4) | | | | 30 | | | (37.5) | | | | | | 25 | | | | (36.2) | | | | | 3 | | | | | (9.7) | | |  |  |  |  |
|  |  |  | I do not care | | | | 32 | | | | (15.1) | | | | 7 | | | | (21.9) | | | | 10 | | | (12.5) | | | | | | 10 | | | | (14.5) | | | | | 5 | | | | | (16.1) | | |  |  |  |  |
| **Female** | |  | |  | | | |  | | | |  | | | |  | | | | BMI categories | | | | |  | | | |  | | | |  | | | |  | | |  | |  | | | |  | | | | | | |
| **Questions** | | **Levels** | | **Total (%)** | | | | | | | | **<18.2(%)** | | | | | | | | **18.2-20.0(%)** | | | | | | | | **20.0-21.8(%)** | | | | | | | | | | **≥21.8(%)** | | | | | | | **p values** | | | | |  | | |
| Have you ever tried to be on a diet | | Yes | | | | 89 | | | | (29.8) | | | | 4 | | | | (9.3) | | | | 28 | | (25.0) | | | | | | 35 | | | | (37.2) | | | | | 22 | | | | (44.0) | | | | p < 0.0006 | | | |  | |
|  |  | No | | | | 210 | | | | (70.2) | | | | 39 | | | | (90.7) | | | | 84 | | (75.0) | | | | | | 59 | | | | (62.8) | | | | | 28 | | | | (56.0) | | | |  |  |  |  |  | |
| Do you want to be slim to be beautiful | | Yes | | | | 186 | | | | (62.0) | | | | 25 | | | | (58.1) | | | | 74 | | (65.5) | | | | | | 55 | | | | (58.5) | | | | | 32 | | | | (64.0) | | | | n.s | | | |  | |
|  |  | No | | | | 114 | | | | (38.0) | | | | 18 | | | | (41.9) | | | | 39 | | (34.5) | | | | | | 39 | | | | (41.5) | | | | | 18 | | | | (36.0) | | | |  |  |  |  |  | |
| How often do you take snacks | | daily | | | | 93 | | | | (31.3) | | | | 14 | | | | (32.6) | | | | 32 | | (28.6) | | | | | | 26 | | | | (28.0) | | | | | 21 | | | | (42.9) | | | | n.s | | | |  | |
|  |  | three or four times per week | | | | 61 | | | | (20.5) | | | | 6 | | | | (14.0) | | | | 30 | | (26.8) | | | | | | 18 | | | | (19.4) | | | | | 7 | | | | (14.3) | | | |  |  |  |  |  | |
|  |  | once or twice per week | | | | 90 | | | | (30.3) | | | | 13 | | | | (30.2) | | | | 30 | | (26.8) | | | | | | 33 | | | | (35.5) | | | | | 14 | | | | (28.6) | | | |  |  |  |  |  | |
|  |  | rarely | | | | 53 | | | | (17.8) | | | | 10 | | | | (23.3) | | | | 20 | | (17.9) | | | | | | 16 | | | | (17.2) | | | | | 7 | | | | (14.3) | | | |  |  |  |  |  | |
| In general, how conscious you are of the | | I want to learn more | | | | 143 | | | | (48.3) | | | | 29 | | | | (69.0) | | | | 46 | | (41.1) | | | | | | 41 | | | | (44.6) | | | | | 27 | | | | (54.0) | | | | p<0.03 | | | |  | |
|  |  | I want to learn in the future | | | | 118 | | | | (39.9) | | | | 8 | | | | (19.0) | | | | 53 | | (47.3) | | | | | | 39 | | | | (42.4) | | | | | 18 | | | | (36.0) | | | |  |  |  |  |  | |
|  | | I am not interested in but better to lern | | | | 28 | | | | (9.5) | | | | 5 | | | | (11.9) | | | | 11 | | (9.8) | | | | | | 7 | | | | (7.6) | | | | | 5 | | | | (10.0) | | | |  |  |  |  |  | |
|  | | I am not interested in this | | | | 7 | | | | (2.4) | | | | 0 | | | | (0.0) | | | | 2 | | (1.8) | | | | | | 5 | | | | (5.4) | | | | | 0 | | | | (0.0) | | | |  |  |  |  |  | |
| Do you consider you should change your | | I want to improve my dietary habits | | | | 167 | | | | (55.9) | | | | 28 | | | | (65.1) | | | | 64 | | (56.6) | | | | | | 48 | | | | (51.6) | | | | | 27 | | | | (54.0) | | | | n.s | | | |  | |
|  |  | I do not want to change | | | | 77 | | | | (25.8) | | | | 10 | | | | (23.3) | | | | 31 | | (27.4) | | | | | | 22 | | | | (23.7) | | | | | 14 | | | | (28.0) | | | |  |  |  |  |  | |
|  |  | I do not care | | | | 55 | | | | (18.4) | | | | 5 | | | | (11.6) | | | | 18 | | (15.9) | | | | | | 23 | | | | (24.7) | | | | | 9 | | | | (18.0) | | | |  |  |  |  |  | |

The body shape perception was compared among BMI category in males and females. A significant difference among BMI groups was determined by Chi-square analyses (p<0.05).
